# Magnetic resonance fingerprinting of the bladder is feasible in men with lower urinary tract symptoms

**DOI:** 10.1007/s00261-026-05428-x

**Published:** 2026-05-02

**Authors:** Aymen Bahsoun, Jesse Hamilton, Giulia Ippolito, Christopher Brace, Chamila Perera, Wilson Sui, Jesus Fajardo, Vikas Gulani, Nicole Seiberlich, Anne Cameron, John Wei, Hero Hussain, Yun Jiang, Shane Wells

**Affiliations:** 1 Department of Radiology, University of Michigan–Ann Arbor, Ann Arbor, USA; 2 Department of Urology, University of Michigan–Ann Arbor, Ann Arbor, USA; 3 Depatment of Radiology, University of Wisconsin–Madison, Madison, USA; 4 Biostatistics Department, University of Michigan–Ann Arbor, Ann Arbor, USA

**Keywords:** Bladder remodeling, Magnetic resonance fingerprinting (MRF), Benign prostate hyperplasia (BPH), Lower urinary tract symptoms (LUTS)

## Abstract

**Purpose:**

To quantify bladder wall relaxation times in men without and with lower urinary tract symptoms (LUTS) using three-dimensional (3D) magnetic resonance fingerprinting (MRF).

**Materials and methods:**

Following informed consent, 51 men (mean age 68.2 years, range 46–80) on active surveillance for prostate cancer underwent multiparametric MRI that included an investigational 3D MRF sequence. Anterior bladder wall T_1_ and T_2_ relaxation times were measured from the quantitative maps. Anterior bladder wall thickness (BWT) was measured and prostate and transition zone (TZ) volumes were estimated, from the conventional T2w images. Body mass index (BMI), LUTS scores (AUA-SI), risk factors for metabolic syndrome, history of diabetes (DM), and medications prescribed for LUTS were documented from the electronic medical record. The Kruskal-Wallis rank sum test, Wilcoxon rank sum exact test, and Fisher’s exact test were used to assess the associations between clinical variables, categorical imaging data, and mean T_1_ and T_2_ relaxation times.

**Results:**

Native bladder wall T_1_ relaxation times were longer in men with higher AUA-SI scores [AUA-SI 0–7: 1,496 ms vs. AUA-SI 8–20: 1,787 ms vs. AUA-SI 21–35: 2,063 ms, *p* = 0.03] while native bladder wall T_2_ relaxation times were similar [AUA-SI 0–7: 67 ms vs. AUA-SI 8–20: 78 ms vs. AUA-SI 21–35: 122 ms, *p* = 0.3]. Native bladder wall T_1_ relaxation times were shorter in diabetic men compared to non-diabetic men (1239 ms vs. 1633 ms, *p* < 0.001), while T_2_ relaxation times were similar (86 ms vs. 62 ms, *p* = 0.4). Prostate and TZ volume, and BWT were similar in men without and with LUTS and DM.

**Conclusion:**

3D MRF of the bladder is feasible, provides in-vivo tissue characterization of the bladder wall in men with LUTS.

## Introduction

Lower urinary tract symptoms (LUTS) are a spectrum of clinical symptoms associated with the storage and elimination of urine that can arise from neurogenic and non-neurogenic etiologies that affect the structure and/or function of the bladder, urethra/sphincter, and prostate. In men, LUTS is frequently attributed to benign prostatic hyperplasia (BPH), a histologic diagnosis that refers to the glandular and stromal (i.e., smooth muscle, connective tissue) proliferation in the prostate transition zone (TZ) that often contributes to an increase in size of the gland. The enlarged prostate can lead to bladder outflow obstruction (BOO), bladder remodeling and the emergence of LUTS, such as urgency, frequency and slow stream. However, it is important to recognize that men without enlarged prostates can develop BOO and LUTS while men with BPH and prostate enlargement may not develop either [1–3].

Lower urinary tract symptoms increase in frequency and severity with age and are non-specific, may be caused by lifestyle choices, medications, and systemic conditions including diabetes and heart failure [1–3]. Further, LUTS affect people differently. Some men with BPH/LUTS and minimal bladder remodeling may be very symptomatic, while other patients with an end-stage bladder may report only mild storage-related symptoms. Unfortunately, the existing non-invasive diagnostic tools used clinically are of limited utility. The anatomic measurements obtained from imaging, including prostate volume (PV), postvoid residual (PVR), and bladder wall thickness (BWT), are only weakly correlated with BOO [4]. While uroflowmetry can identify patients with low urine flow, it is unable to reliably differentiate BOO, detrusor underactivity, and other causes of a slow stream. All of this complicates the diagnosis of BOO and appropriate risk stratification. As a result, men with long-standing BOO and advanced bladder remodeling, in need of urgent outlet surgery, are often trialed to medical therapy where the therapeutic window to preserve bladder function may be lost.

Magnetic resonance fingerprinting (MRF) is a multiparametric MRI technique that acquires temporal signatures (“fingerprints”) instead of traditional images [5–9]. These fingerprints are matched to a precomputed dictionary, derived from physics simulations, to generate quantitative images, or maps, of intrinsic tissue properties. This method, frequently used in cardiac imaging, maps MRI-specific tissue properties, including T_1_ (chronic inflammation), T_2_ (acute inflammation and edema), extracellular volume fraction (alterations in the interstitial space), and proton density (M_0_) (anatomy), among others [6, 7, 10–12]. Recent advances in MRF, including the development of three dimensional (3D) techniques, provide volumetric maps with high-resolution imaging capable of characterizing the bladder wall [13]. Unique combinations of these tissue properties, or bladder wall signatures, in patients with LUTS may reveal distinct phenotypes and provide quantitative, non-invasive and reproducible metrics that may guide treatment selection and response to therapy. Therefore, the purpose of this study was to quantify bladder wall T_1_ and T_2_ relaxation times in men without and with LUTS using 3D MRF.

## Materials and methods

This prospective, single-center, cross-sectional study was IRB-approved and HIPAA compliant. Informed consent was obtained from all participants. Men with prostate cancer on active surveillance scheduled to undergo multiparametric MRI (mpMRI) of the pelvis were recruited to participate in this study between 7/2022 and 6/2024.

### MRI and MRF acquisition

A 3 T mpMRI of the pelvis using a 32-channel phased array coil was performed on all participants (MAGNETOM Vida, Siemens Healthineers, Erlangen, Germany). Participants voided immediately prior to mpMRI. The mpMRI included the following acquisitions: axial 2D and 3D T2w TSE, coronal and sagittal 2D T2w TSE, axial DWI (b100, b800 and b1600), axial T1w fat saturation, and dynamic contrast enhanced (DCE) axial T1w fat saturation following intravenous bolus administration of a gadolinium-based contrast agent (gadobenate dimeglumine 0.1 mmol/kg (Table 1).

**Table 1:** Multiparametric MRI protocol of the pelvis at 3 T (MAGNETOM Vida, Siemens Healthineers)

Sequence	TR (ms)	TE (ms)	Acquisition time (sec)	Spatial resolution (mm)	Matrix size	FOV (mm)	Slice thickness (mm)	Averages
Coronal T2w TSE	7000	107	2:06	.3 × 3 × 3	334 × 384	200 × 200	3	1
Sagittal T2w TSE	6640	107	1:46	.3 × 3 × 3	288 × 384	200 × 200	3	1
Axial T2w TSE	6640	107	1:59	0.3 × 0.3 × 3.0	334 × 384	200 × 200	3	1
Axial 3D T2w SPACE	1500	153	4:48	0.6 × 0.6 × 1.0	272 × 320	200 × 200	1	1.4
DWI (b 0, 100, 800, 1600)	3500	92	7:40	0.8 × 0.8 × 4.0	114 × 114	200 × 200	4	1, 4, 6, 22
T1w FS (pre)	4.43	TE1: 1.54 TE2: 2.46	0:21	1.2 × 1.2 × 3	195 × 320	380 × 81.1	3	1
DCE T1w FS	3.89	1.40	3:14	.9x.9 × 1.8	102 × 128	220 × 84.4	1.8	1

T2-weighted (T2w), T1-weighted (T1w), Diffusion-Weighted Imaging (DWI), Dynamic Contrast Enhancement (DCE), Turbo Spin Echo (TSE), Sampling Perfection with Application-optimized Contrasts using different flip angle Evolution (SPACE)

A 3D MRF acquisition based on the Fast Imaging with Steady-state Precession (FISP) sequence with a 3D stack-of-spirals trajectory and 48x under sampling with 70% partial Fourier was implemented with a factor of 0.7 along the partition dimension and a k-t under sampling of 3 along the temporal dimension, to achieve a resolution of 1 × 1 × 1 mm^3^ in 7.5 min with a 400 × 400 × 60 mm^3^ field-of-view centered on the prostate [14, 15]. Image reconstruction was performed offline in Matlab (MathWorks, Natick, MA, USA). The Projection Onto Convex Sets (POCS) method was used to reconstruct the k-space missing phase from the PF acquisition. The data were gridded using non-uniform fast Fourier transform. Image blurring was corrected by applying conjugate phase reconstruction using a pre-acquired B_0_ map. A dictionary containing 541,890 entries was calculated using Bloch Equations with T_1_ values between 10 and 5000 ms, T_2_ values between 2 and 800 ms, and scaling factors of (actual/nominal) B_1_+ values from 0.6 to 1.4. The B_1_+ inhomogeneities were obtained from a pre-acquired B_1_+ map [14–16].

### Data collection

Clinical and imaging data were collected from the electronic medical record and picture archiving and communication system (PACS) (SECTRA 25.2, Linköping, Sweden). Images and T1 and T2 maps obtained from the MRF data were displayed using a DICOM viewer (Osirix MD 14.1.1, Geneva, Switzerland).

### Clinical data

The clinical variables obtained, from the electronic medical record, included age, body mass index (BMI), and risk factors for metabolic syndrome including hypertension (HTN), diabetes mellitus (DM), and abnormal lipid profile [elevated triglycerides (TG), low high-density lipoprotein (HDL)]. Abdominal circumference measurements were not available. Participants were considered to have metabolic syndrome when three of the remaining four diagnostic criteria coexisted (HTN, DM, low HDL, high TG). The American Urological Association symptom index (AUS-SI) and quality of life (QOL) scores were recorded. The use of medications for LUTS [alpha blockers, 5α-reductase inhibitors (5ARI), and phosphodiesterase-5 inhibitors (PDE5)] was documented. For this study, only the 5 mg daily dose of PDE5 daily was considered medical therapy for LUTS.

### Imaging data

Prostate and transition zone (TZ) volumes were estimated from the ellipsoid formula using the transverse, anterior-posterior, and longitudinal linear measurements obtained from the axial and sagittal T2w MRI. Anterior bladder wall thickness was measured on the axial T2w MRI.

**Fig. 1:**
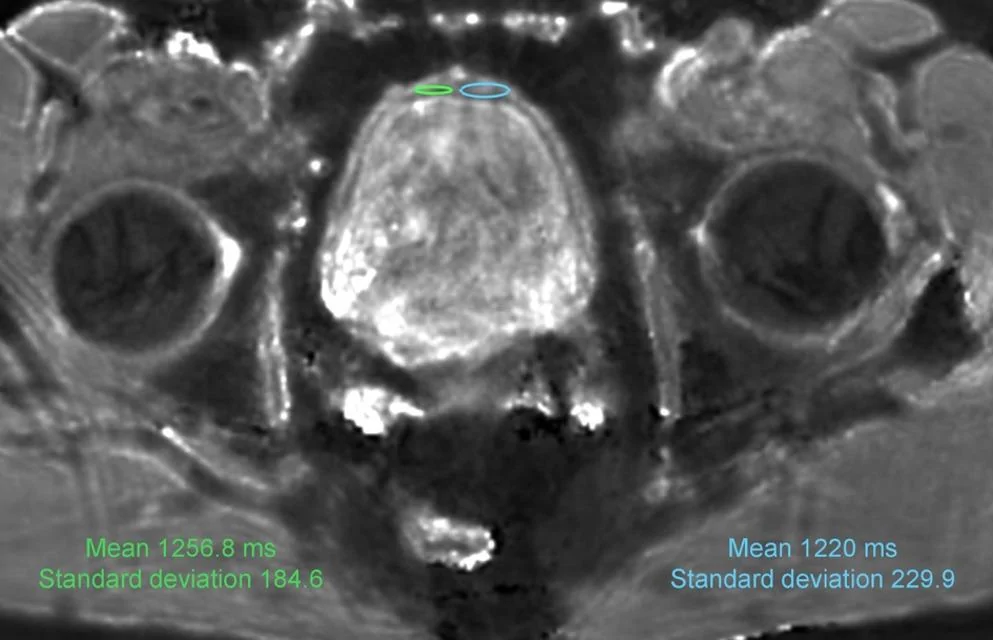
Quantitative T1 map of the pelvis acquired using an investigational 3D MR Fingerprinting (MRF) sequence on a 3 T scanner (MAGNETOM Vida, Siemens ddHealthineers). Bladder wall T_1_ relaxation time was averaged from two regions-of-interest placed on the anterior bladder wall

Two freehand, regions-of-interest (ROI) were placed on the para-midline right and left anterior bladder wall of the axial T_1_ and T_2_ maps (Fig. 1) by a postdoctoral medical student under the supervision of an abdominal radiologist with 15 years of prostate/bladder MRI experience. The oval-shaped ROIs were drawn to include the entire thickness of the bladder wall while avoiding the perivesical fat, urine in the bladder, and obvious artifacts. Mean bladder wall T_1_ and T_2_ relaxation times were calculated from the two ROIs and recorded.

### Statistical analysis

Continuous features are summarized with mean and standard deviation (SD) or median and interquartile range (IQR). Categorical data are summarized with frequency counts and percentages. The Kruskal-Wallis rank sum test, Wilcoxon rank sum exact test, and Fisher’s exact test were used to assess the associations between clinical variables, categorical imaging data, and mean T_1_ and T_2_ relaxation times. All statistical analyses were performed using R Version 4.3.1. *p* < 0.05 was considered significant for all statistical tests.

## Results

### Clinical data

Fifty-one men were recruited to participate in this pilot imaging study. Participants had a mean age was 68.2 years (range 46–80), a median BMI of 27.2 (IQR 25.2–30.4), and coexisting comorbidities including: HTN (62%, 32/51), abnormal lipid profile (80%, 41/51), and DM (14%, 7/51).

Thirty-eight men (75%) had a documented AUA-SI score with a median AUA-SI of 12 (IQR: 8.5–16) and QOL index of 2 (IQR: 2–3). Twenty-nine (57%) men were on medical therapy for LUTS including 17 men (59%, 17/29) on single drug therapy and 11 men (38%) on combination therapy. Men on medical therapy were prescribed alpha blockers (24/29, 83%), PDE5s (12/29, 41%) and/or 5ARIs (5/29, 17%).

### Imaging data

Median prostate and TZ volumes were 104 mL (IQR: 71–127) and 28 mL (IQR: 24–52). Median anterior BWT was 6 mm (IQR: 5–7). Prostate and TZ volume, and anterior BWT were similar across BMI (*p* = 0.9), AUA-SI score categories (mild, moderate, and severe) (*p* = 0.4–0.6), and among men prescribed and not currently prescribed medications for LUTS (*p* = 0.5–0.9), and Tables 2, 3 and 4.

**Table 2:** Quantitative prostate and bladder anatomic and MR Fingerprinting data stratified by participant body mass index

Characteristic	Normal *N* = 12^1^	Overweight *N* = 22^1^	Obese *N* = 17^1^	*p*-value^2^
Prostate volume (mL)	83 (59–147)	109 (69–130)	104 (79–110)	0.9
Transition zone volume (mL)	29 (24–64)	28 (25–49)	30 (22–46)	> 0.9
Bladder wall thickness (mm)	5.30 (4.45–7.98)	5.84 (4.45–7.15)	5.80 (4.90–7.14)	> 0.9
Bladder wall T1 (ms)	1,480 (1,333-2,106)	1,587 (1,356-1,992)	1,633 (1,244-2,024)	0.8
Bladder wall T2 (ms)	63 (36–78)	81 (32–117)	64 (40–92)	> 0.9

^1^Median (Q1–Q3), ^2^Kruskal-Wallis rank sum test

**Table 3:** Quantitative prostate and bladder anatomic and MR Fingerprinting data stratified by men prescribed (yes) and not currently prescribed medical therapy for LUTS

Characteristic	Yes *N* = 29^1^	No *N* = 22^1^	*p*-value^2^
Prostate volume (mL)	104 (83–116)	97 (64–156)	> 0.9
Transition zone volume (mL)	28 (23–44)	29 (25–64)	0.5
Bladder wall thickness (mm)	5.30 (4.80–7.14)	5.90 (4.45–7.40)	0.7
Bladder wall T_1_ (ms)	1,817 (1,356-2,024)	1,494 (1,316-1,866)	0.4
Bladder wall T_2_ (ms)	64 (39, 86)	74 (35, 110)	0.5

^1^Median (Q1-Q3), ^2^Wilcoxon rank sum exact test

**Table 4:** Quantitative prostate and bladder anatomic and MR Fingerprinting data stratified by AUA-SI score

Characteristic	Mild *N* = 16^1^	Moderate *N* = 19^1^	Severe *N* = 3^1^	*p*-value^2^
Prostate volume (mL)	100 (69, 147)	105 (73, 130)	70 (47, 95)	0.4
Transition zone volume (mL)	29 (24, 61)	28 (25, 49)	22 (22, 28)	0.4
Bladder wall thickness (mm)	6.83 (4.48, 8.30)	5.68 (4.87, 6.80)	4.90 (4.80, 8.00)	0.6
Bladder wall T_1_ (ms)	1,385 (1,282, 1,631)	1,542 (1,307, 2,024)	2,063 (1,999, 2,483)	0.03
Bladder wall T_2_ (ms)	67 (46, 101)	78 (33, 92)	122 (70, 158)	0.3

^1^Median (Q1-Q3), ^2^Kruskal-Wallis rank sum test

**Fig. 2:**
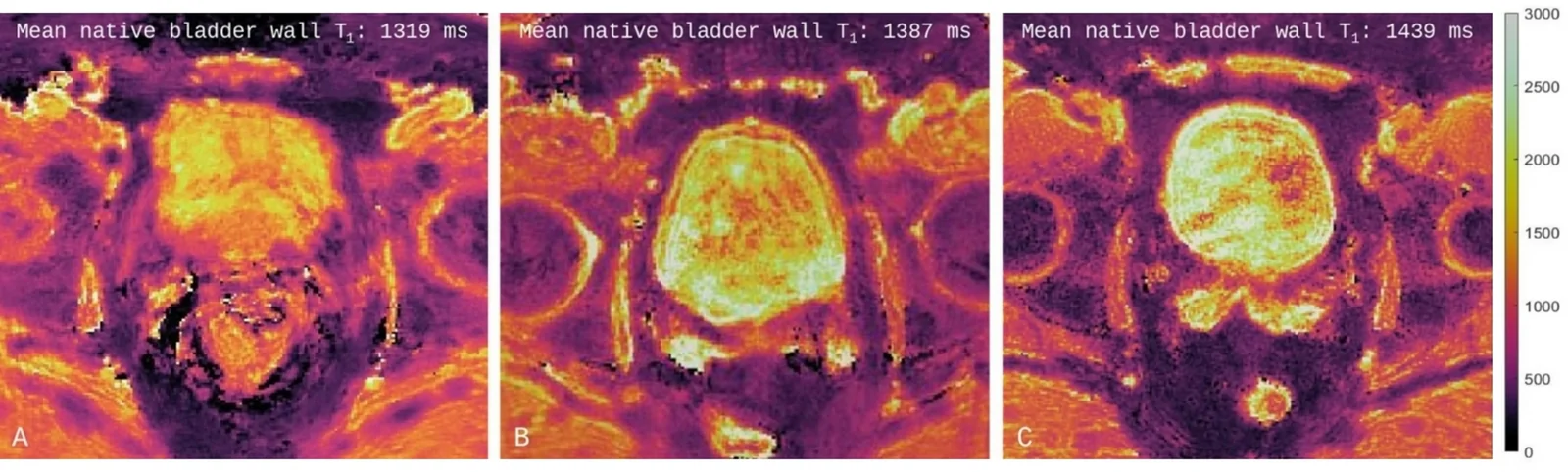
Representative T_1_ color maps from Magnetic Resonance Fingerprinting (MRF) in a 61-year-old man with mild lower urinary tract symptoms (LUTS) (AUA-SI 6) (**A**), a 74-year-old man with moderate LUTS (AUA-SI 12) (**B**), and a 62-year-old man with severe LUTS (AUA-SI 27) (**C**). Native bladder wall T_1_ were longer in men with higher lower urinary tract symptom scores

Median native bladder wall T_1_ and T_2_ relaxation times were 1574 ms (IQR: 1336–1999) and 66 ms (IQR: 37–98), respectively. Bladder wall T_1_ and T_2_ relaxation times were similar across BMI (*p* = 0.8–0.9) and among men prescribed and not currently prescribed medications for LUTS (*p* = 0.4–0.5). (Tables 2 and 3) Men with more bothersome LUTS, as stratified by the AUA-SI, into mild (AUA-SI: 0–7), moderate (AUA-SI: 8–20), and severe (AUA-SI: 21–35) categories, had progressively longer native bladder wall T_1_ relaxation times [1,385 ms (IQR: 1,282-1,631) vs. 1,542 ms (IQR: 1,307-2,024) vs. 2,063 ms (IQR: 1,999-2,483), *p* = 0.03] while native bladder wall T_2_ relaxation times were similar [67 ms (IQR: 46–101) vs. 78 ms (IQR: 33–92) vs. 122 ms (70–158), *p* = 0.3]. (Table 4) (Fig. 2).

**Fig. 3:**
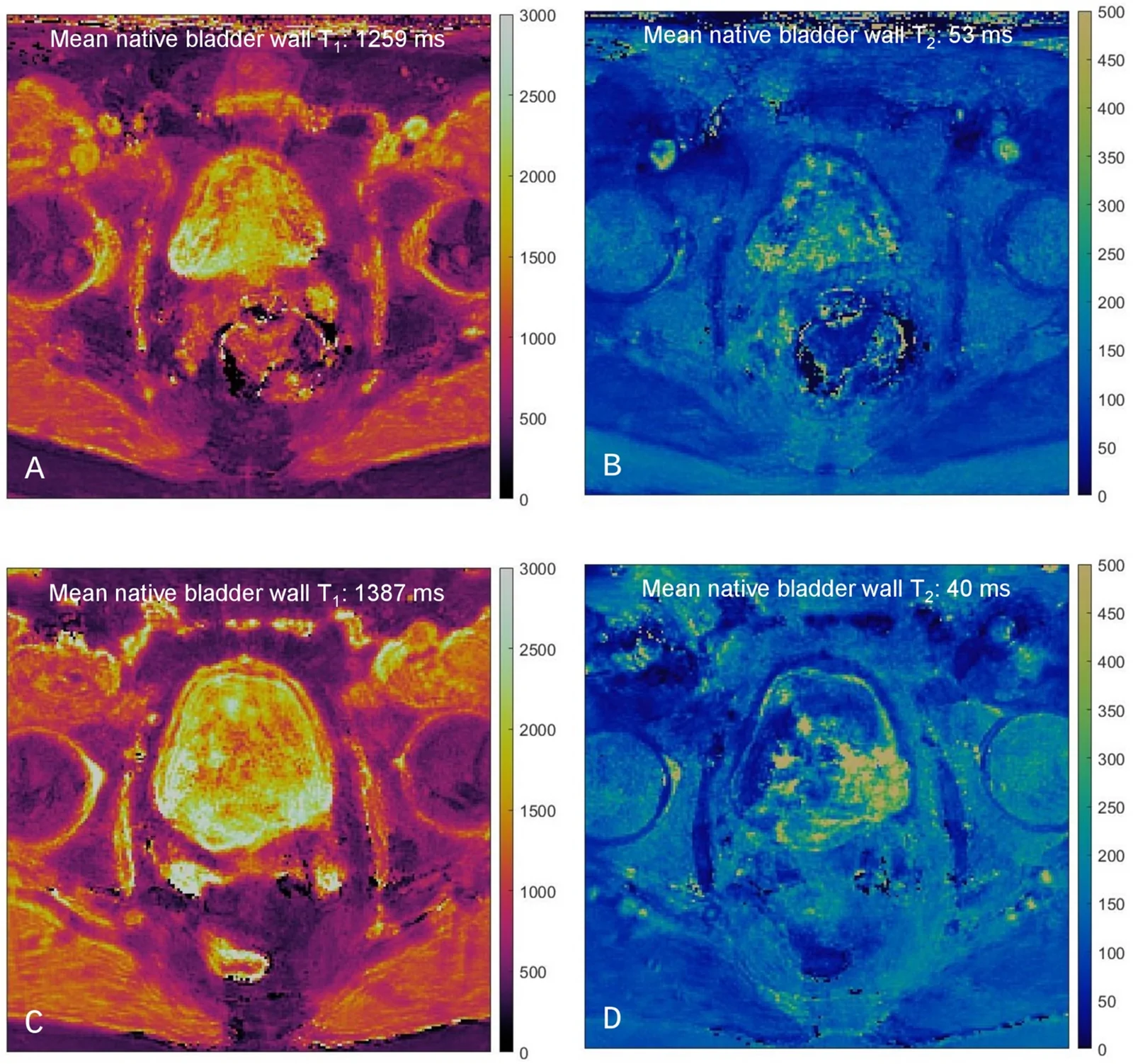
Representative T_1_ (**A**, **C**) and T_2_ (**B**, **D**) color maps from Magnetic Resonance Fingerprinting (MRF) in a 72-year-old man with diabetes mellitus (top row) and a 61-year-old man (bottom row) without diabetes. Native bladder wall T_1_ was shorter in diabetic men compared to non-diabetic men, while bladder wall T_2_ was similar

While diabetic and non-diabetic men were similar in age (72 vs. 70 years, *p* = 0.8), they were more likely to be obese (5/7, 71% vs. 12/44, 28%; *p* = 0.03) and have metabolic syndrome (5/7, 71% vs. 5/44, 11%; *p* = 0.002). LUTS severity (mean AUA-SI: 10 vs. 9.7, *p* = 0.59) and number of men prescribed medical therapy for LUTS (5/7, 71% vs. 24/44, 56%, *p* = 0.25) was similar in diabetic and non-diabetic men. The prostate volume, TZ volume, and anterior BWT were similar in men without and with DM (*p* = 0.5–0.7). Bladder wall T_1_ relaxation times were shorter in diabetic men compared to non-diabetic men (1239 ms vs. 1633 ms, *p* < 0.001), while bladder wall T_2_ relaxation times were similar (86 ms vs. 62 ms, *p* = 0.4). (Table 5) (Fig. 3).

**Table 5:** Quantitative clinical, prostate and bladder anatomic and MR Fingerprinting data in men with and without diabetes mellitus

Characteristic	Yes *N* = 7^1^	No *N* = 44^1^	*p*-value^2^
Age^3^ (years)	72 (61–73)	70 (622 − 75)	0.8
BMI^3^ (kg/m^2^)	31.8 (25.7, 34.9)	27.2 (25.2, 30.4)	0.09
MetabolicSyndrome^**2**^			0.002
Yes	5 (71%)	5 (11%)	
No	2 (39%)	39 (79%)	
AUA SI score^3^	10 (3–14)	9 (8–9)	0.588
Prostate volume (mL)	106 (69–115)	104 (71–128)	0.5
Transition zone volume (mL)	29 (25–57)	26 (20–46)	0.7
Bladder wall thickness (mm)	5.80 (5.1–7.25)	5.59 (4.43–7.15)	0.7
Bladder wall T1 (ms)	1,239 (1,145-1,259)	1,633 (1,365-2,031)	< 0.001
Bladder wall T2 (ms)	86 (57–92)	62 (34–107)	0.4

^1^Median (Q1-Q3), ^2^Wilcoxon rank sum test, ^3^Fisher’s exact test

## Discussion

There is growing interest in quantitative MRI, including MRF, which can provide maps whose pixel values are absolute measurements of intrinsic tissue properties. Commonly measured tissue properties include T_1_ and T_2_ relaxation times, which describe how water protons behave in a magnetic field. These properties are clinically relevant because different tissues have characteristic normal ranges with deviations indicating the presence of pathology. The overarching goal of this study was to quantify bladder wall T_1_ and T_2_ relaxation times in men without and with LUTS. We observed longer native bladder wall T_1_ relaxation times in men with worsening LUTS, as stratified by the AUA-SI. We hypothesize that longer T_1_ relaxation times suggests the presence of higher bladder wall collagen content (i.e., fibrosis) [8]. We also found that men with diabetes had shorter native bladder wall T_1_ relaxation times than men without diabetes. We hypothesize that shorter T_1_ relaxation times, observed in this study, suggest the presence of higher bladder wall fat content rather than edema/inflammation, which would result in a long T_2_ relaxation time alongside short T_1_ relaxation time [11]. This observation could be confirmed by adding another quantitative parameter, proton density fat fraction (PDFF), to the bladder wall signature map. Importantly, men in both cohorts had similar prostate and TZ volumes and BWT, supporting prior studies suggesting that these often-used clinical features are of limited independent utility in the assessment of men with LUTS [17].

T_1_ mapping is an accurate, reproducible, and non-invasive method to quantify the spatiotemporal distribution of myocardial fibrosis (i.e. collagen deposition) in men with aortic stenosis and correlates with function (i.e., stroke volume, ejection fraction) and prognosis [18–20]. Similar to the left ventricle, in the setting of aortic stenosis, the bladder responds to outlet resistance due to BOO across three stages: hypertrophy, compensation, and decompensation [3]. Detrusor smooth muscle hypertrophy occurs in response to cyclic mechanical stress (high voiding pressure) from BOO. The resulting increased oxygen demand coupled with reduced delivery contribute to detrusor hypoxia. Persistent hypoxia activates signaling pathways that alter the extracellular matrix resulting in the production and deposition of collagen. When the detrusor can no longer compensate for the high cyclical pressures during voiding, the downward spiral towards detrusor underactivity (DU) begins [21–23]. Diabetes alters microvascular perfusion accelerating oxidate stress, hypoxia, and fibrosis [24]. Finally, bothersome LUTS emerge and fluctuate during these responses to BOO.

In this study, the observed longer native T_1_ relaxation times in the anterior bladder wall of men with worsening AUA symptom index scores support the hypothesize that BOO contributes to bladder wall remodeling and the development of bladder wall fibrosis. This remodeling process is probably regionally heterogeneous. Specific segments of the bladder wall, such as the posterior dome, may be affected earlier and more severely while other segments of the bladder, such as the trigone, may be relatively sparred. Therefore, ROIs placed on the anterior bladder wall may not fully represent the extent of bladder wall remodeling. An optimized 3D MRF sequence that provides full bladder coverage can be used in future studies and deployed on participant cohorts that include aging men without and with symptoms with ROIs placed on the regionally segmented bladder wall to assess the spatial distribution of T_1_ and T_2_ relaxation times. Urodynamic testing will also need to be incorporated into future studies so that bladder wall relaxation times can be correlated with detrusor function. Inflammation and fibrosis alter bladder wall compliance and detrusor contractility. Mapping the extent of change in bladder wall T_1_ and T_2_ relaxation times against detrusor function will be critical for translating this technique into clinical practice. Men with suspected BOO and high native bladder wall T_1_ relaxation times could be triaged to early surgery, regardless of symptom scores, mitigating the risk for DU, while men with suspected BOO and normal native bladder wall T_1_ relaxation times may have a wider therapeutic window to trial medical therapy.

This prospective, cross-sectional study highlights the potential power of translating quantitative MRI techniques to imaging of the bladder wall but has several important limitations that require additional investigation. Lack of normative data limit the conclusions that can be drawn from the reported bladder wall relaxation times. While the study population which was representative of a typical BPH cohort, the patients were on active surveillance for prostate cancer. Bladder wall tissue signatures may change with aging even in the absence of disease, in response to normal hormonal variation, and with medical therapies for other disease, including prostate cancer. The prospective design is a strength; however, a longitudinal rather than cross-sectional approach would likely provide additional insights into the temporal evolution of bladder wall relaxation times in men with longstanding BOO. While the study population was sufficient to demonstrate feasibility and explore relationships between LUTS and bladder wall relaxation times, it was underpowered for LUTS and diabetes subgroup analysis. Clinically relevant data, such as AUA-SI scores were not available for all participants and medication adherence information was not available for any participant. Lastly, the duration of diabetes and level of glycemic control are known to contribute to DBD [24]. Application of 3D MRF to a well-studied, longitudinal cohort such as the participants in The Epidemiology of Diabetes Interventions and Complications (EDIC) study would be extremely valuable.

In conclusion, 3D MRF of the bladder is feasible, provides in-vivo tissue characterization of the bladder wall in men with LUTS. When validated, we anticipate that patients with LUTS will cluster into distinct phenotypes within a multidimensional space defined by unique combinations of bladder wall signatures (T_1_, T_2_, PDFF), anatomical measurements (PV, BWT, PVR), and clinical data (AUA-SI, UDS) providing a robust framework for diagnosing and monitoring treatment response that shifts the paradigm away from the current trial-and-error approach towards personalized care.

## Data Availability

All data supporting the findings of this study are available within the paper.
